# Supplementary material for: Fundamental limits on inferring epidemic resurgence in real time using effective reproduction numbers
Source: PLoS Comput Biol. 2022 Apr 11;18(4):e1010004. doi: 10.1371/journal.pcbi.1010004 (PMC9022826; doi:10.1371/journal.pcbi.1010004)
Supplement: S1 Appendix — Fig A: Resurgence and control dynamics of COVID-19. We repeat the simulations from Fig 2 but for realisations of COVID-19 epidemics. Fig B: Incidence curves for COVID-19. We present the simulated counts of daily new cases that underlie the results of Fig A. Fig C: Incidence curves for Ebola virus disease. We present the counts of daily new cases that underlie the results of Fig 2 of the main text. (PDF) [file pcbi.1010004.s001.pdf]

S1 Appendix

Fundamental limits on inferring epidemic resurgence in real time using effective reproduction numbers – Kris V Parag and Christl A Donnelly.

We provide simulations in **Fig A** for simulated COVID-19 epidemics, showing that significant delays in detecting resurgence but not epidemic control persist. These are consistent with and support **Fig 2** of the main text, which examined Ebola virus disease dynamics. While figures plot ensembles of mean estimates, single simulations (where the estimated credible intervals reflect noise from the incidence of that simulation) also display this asymmetry, confirming that real-time resurgence detection is innately hard.

We also provide plots of the simulated incidence curves (i.e., daily counts of infected cases), which underlie the results of **Fig A** above and **Fig 2** of the main text, in **Fig B** and **Fig C** respectively. The generally smaller incidence (which is also stochastically noisier) that often associates with resurgence events contributes to their innate detection difficulty.

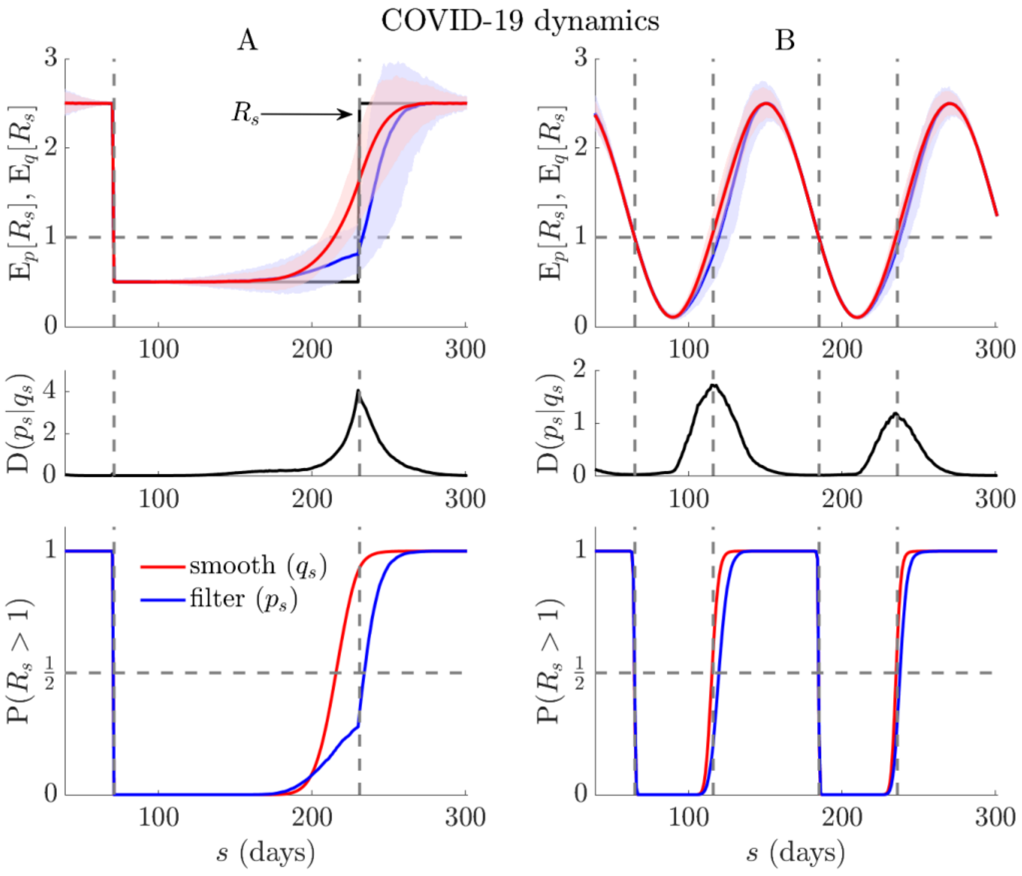

Fig A: Resurgence and control dynamics of COVID-19. We repeat the simulations from **Fig 2** (main text) but for 1000 realisations of COVID-19 epidemics ($t = 300$) using generation times from [1] (true R_s in black). Top panels show the posterior mean estimates from every realisation (computed via *EpiFilter* [2]). Middle panels average Kullback-Liebler divergences from those simulations, $D(p_s|q_s)$, and bottom panels show the overall filtered ($\mathrm{P}(R_s > 1 \mid I_1^s)$, blue), and smoothed ($\mathrm{P}(R_s > 1 \mid I_1^t)$, red) probabilities of resurgence. In keeping with **Fig 2**, we find fundamental and appreciable latencies in detecting resurgence, often an order of magnitude longer than those for detecting epidemic control (compare red and blue curves in relevant panels). The initial rise in $\mathrm{P}(R_s > 1 \mid I_1^s)$ of panel A, which precedes the R_s change is due to the prior distribution over R_s (which has mean > 1) in a period with very few cases.

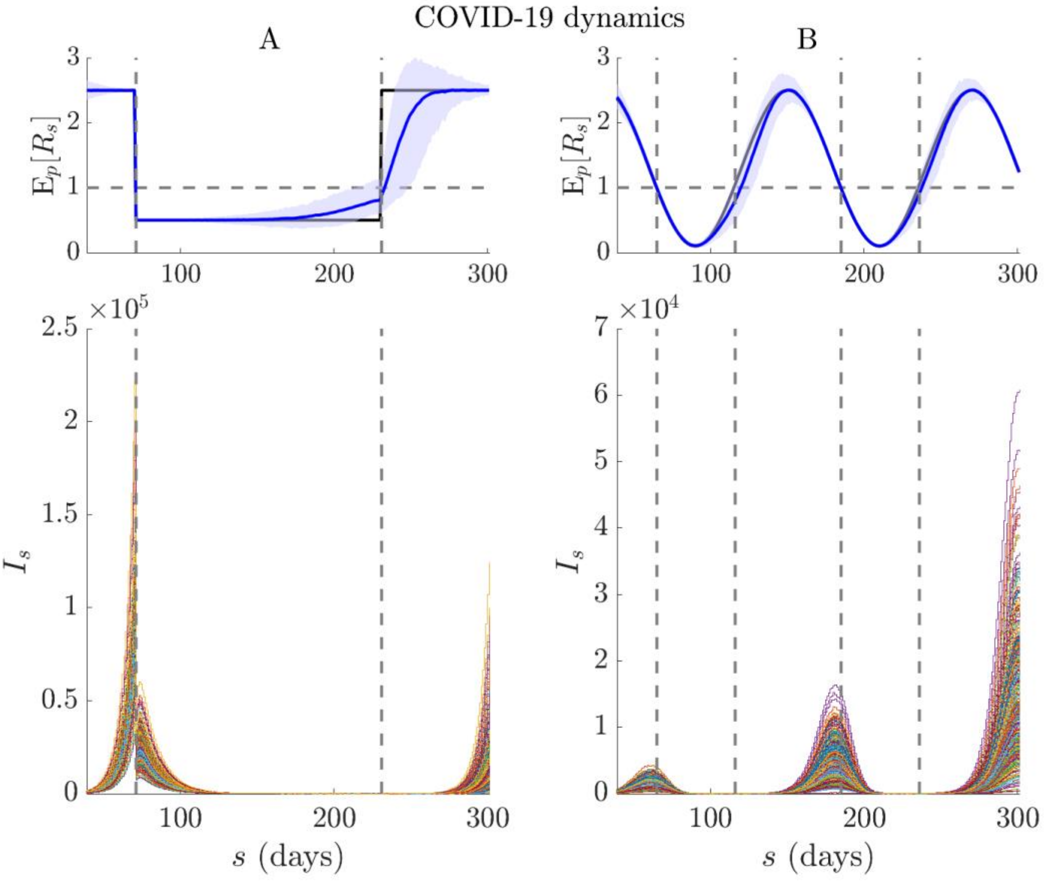

Fig B: Incidence curves for COVID-19. We present the counts of daily new cases, I_s, (bottom panels), which are simulated from the R_s trajectories (black, top panels) depicting a rapid control and resurgence (A) and a seasonally varying transmissibility (B). These curves are generated using renewal models (see Methods above) with COVID-19 generation times from [1] and underlie the results in **Fig A**. We include the filtered (real-time) estimates, $\mathrm{E}_p[R_s]$, from that figure for completeness (blue with 95% credible intervals).

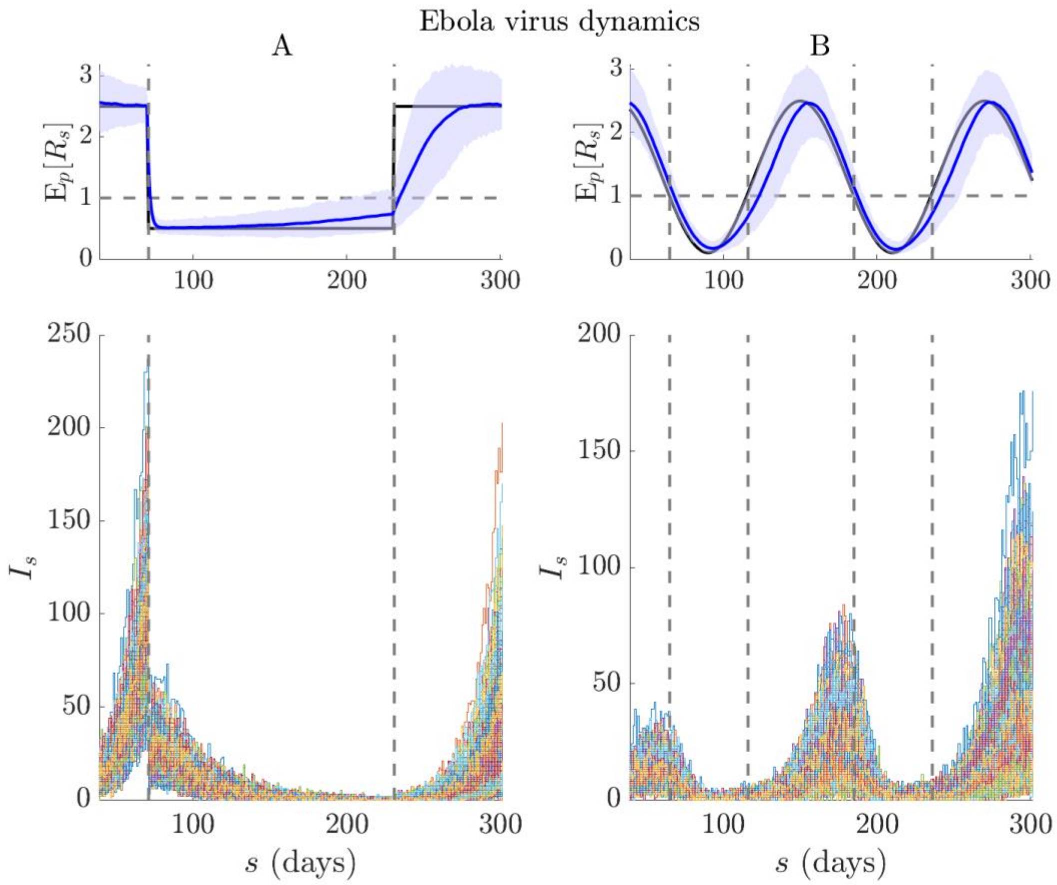

Fig C: Incidence curves for Ebola virus disease. We present the counts of daily new cases, I_s, (bottom panels), which are simulated from the R_s trajectories (black, top panels) depicting a rapid control and resurgence (A) and a seasonally varying transmissibility (B). These curves are generated using renewal models (see Methods above) with Ebola virus generation times from [3] and underlie the results in **Fig 2** of the main text. We include the filtered (real-time) estimates, $\mathrm{E}_p[R_s]$, from that figure for completeness (blue with 95% credible intervals).

References

1. Ferguson N, Laydon D, Nedjati-Gilani G, Others. Impact of non-pharmaceutical interventions (NPIs) to reduce COVID- 19 mortality and healthcare demand. Imperial College London; 2020.
2. Parag KV. Improved estimation of time-varying reproduction numbers at low case incidence and between epidemic waves. PLoS Comput Biol. 2021;17: e1009347. doi:10.1371/journal.pcbi.1009347
3. Van Kerkhove MD, Bento AI, Mills HL, Ferguson NM, Donnelly CA. A review of epidemiological parameters from Ebola outbreaks to inform early public health decision-making. Sci Data. 2015;2: 150019. doi:10.1038/sdata.2015.19
