# Supplementary material for: Fundamental limits on inferring epidemic resurgence in real time using effective reproduction numbers
Source: PLoS Comput Biol. 2022 Apr 11;18(4):e1010004. doi: 10.1371/journal.pcbi.1010004 (PMC9022826; doi:10.1371/journal.pcbi.1010004)
Supplement: Attachment — Submitted filename: Parag_review_upload.pdf [file pcbi.1010004.s002.pdf]

In "Fundamental limits on inferring epidemic resurgence in real time using effective reproduction numbers" by Parag and Donnelly, the authors adopt a renewal model for epidemic progression and consider inference on the time-varying reproduction number R_s in a growing/resurging or declining/controlled epidemic. The authors argue that there is an inherent asymmetry in the ability to detect a growing epidemic in realtime that arises due to the limited information that can be extracted from low case numbers. Parag & Donnelly argue that even with state-of-the-art Bayesian optimisation in practice this means that there is a lag of around a generation time before a shift to R_s exceeding its threshold value can be detected, even with perfect case reporting, for a selection of 5 pathogens with different generation times. The authors further demonstrate that this delay in detecting resurgence is exacerbated by aggregating data across any heterogeneity.

This manuscript makes some interesting points that are important considerations for developing surveillance systems that aim to detect early outbreaks. The discussion of alternative and composite surveillance techniques is also valuable.

Some minor points below:

As noted the asymmetry in detecting resurgence is due to the assumption that if R_s rises above 1 this is likely at a time when the epidemic is small (with small $\Lambda_{\tau(s)}$). During the SARS-CoV-2 pandemic we have sometimes seen R_s increase above at a time when the epidemic is large, so it is perhaps worth noting that this less likely combination of $\lambda_{\tau(s)}$ and $\Delta\lambda_{\tau(s)}$ may still occur, and discuss whether case detection could still provide a relatively efficient and valuable route to inference of R_s in these situations?

The decision to explore inference of R_s in the case of perfect case detection is well motivated. As I understand it this analysis also assumes perfect knowledge of the generation interval distribution w_u, which may change over time for many reasons. Changes in R_s due to shifts in behaviour (Ali et al., 2020) or due to emergence of new variants (Hart et al., 2021) may in particular be accompanied by changes in w_u. It could be interesting to discuss whether the asymmetry in sensitivity between detection of control and resurgence would still be significant when considering other such sources of uncertainty.

Ali et al., 2020, https://pubmed.ncbi.nlm.nih.gov/32694200/

Hart et al., 2021, https://www.medrxiv.org/content/10.1101/2021.10.21.21265216v1
