# Supplementary material for: Fundamental limits on inferring epidemic resurgence in real time using effective reproduction numbers
Source: PLoS Comput Biol. 2022 Apr 11;18(4):e1010004. doi: 10.1371/journal.pcbi.1010004 (PMC9022826; doi:10.1371/journal.pcbi.1010004)
Supplement: Attachment — Submitted filename: responses.docx [file pcbi.1010004.s003.docx]

**Responses to Reviewers: PCOMPBIOL-D-21-02218**

We thank the reviewers for their constructive and insightful comments. Our responses to each point and corresponding changes to the main text, are listed below. We include both clean and tracked-changes versions of the revised text.

**Reviewer #1:**

In "Fundamental limits on inferring epidemic resurgence in real time using effective reproduction numbers", Parag and Donnelly examine our ability to detect changes in epidemic growth, as characterized by the reproductive number R, in real time. They find that there are delays in our ability to detect changes in the reproductive number, particularly changes around the critical threshold of R being above or below 1, that are inherent in the generating process and hence cannot be overcome by improved analysis of epidemic curves. In many ways their results are obvious, shifts in R cannot be detected without data, and since that data comes from subsequent generations of infection that are necessarily delayed by the generation time of the disease, there is an upper limit to how quickly we can detect these shifts. Likewise, as more cases are present when epidemics shift to a decline than when they shift to a resurgence, statistical theory tells us the former will be easier to detect than the latter.

My statement that these conclusions are in retrospect obvious is in no way a negative comment on this work…much of the best science seems obvious in retrospect. The thorough and comprehensive way in which the authors prove their point and quantify many of these delays is compelling and invaluable, and I found no technical problems with their work. I found the use of classical statistical measures, such as Fisher information, to characterize many of the results a particularly compelling. Hence, I think this analysis is a strong and important contribution to the literature.

Many thanks for the positive appraisal.

As detailed below, I do think the presentation (particularly the figures) could be made more clear and that the authors could be a little bit more nuanced in their discussion of the practical impact of these results. However, these are simply improvements to what is already an interesting and informative paper.

As listed below, we have aimed to amend the script in line with these clarity and nuance issues. Specifically, we have added more context in the Discussion and reworked the Figure labels and captions to better guide the reader.

Specific Comments:

1 Abstract, "This belies epidemic…"
I don't think this means quite what the authors intend, or I am misunderstanding the rest of the sentence.

Thanks for pointing this out, we have replaced ‘This belies’ with ‘In contrast’. We simply meant to highlight the asymmetry between detecting control and resurgence here.

2 Abstract, "Responses to recrudescent…"
I think this misses the nuance that is around a similar statement in the main text. I.e., it does not come across that what is being argued is that improvements to analysis of epidemics curves are less important than improving surveillance.

We have rewritten this line to state this point more explicitly.

3 Introduction, "…enhancing syndromic…Such systems…"
I feel like the argument here is broader than syndromic surveillance, as highlighted by the reference to wastewater surveillance later. I.e., the authors appear to be arguing that novel methods beyond syndromic surveillance might be useful.

We have removed the term ‘syndromic’ and reworded the text to indicate that what we are really proposing as a solution is a more holistic use of multiple data sources.

4 Figures 1-4
I will admit to finding the figures a bit hard to decode, and wonder if something can be done to make the meaning of the colors, axes, etc. a bit more clear.

Apologies for the nebulous figures. We have reworked all figures, with additional labels and legends to indicate key parameters or variables. We have tried to keep a fixed colour scheme whenever we vary a parameter (red is high, grey intermediate and blue is low). We have also updated all captions with more explicit descriptions of notation. Hopefully the figures and their main points are now more intelligible.

5 Discussion, "…there are grounds….that enact interventions…at the expense of false alarms"
I wonder if this argument goes too far beyond what the analysis in the paper supports, and might warrant a more nuanced discussion. Without an analysis of the cost of false alarms, it is hard to say if a conservative approach is warranted or not. Further, this decision will depend on the cost of an intervention over the short and long term, which will likely be specific to intervention and context. As to the latter point, my perception is that in the United States public health agencies had the political capital to implement interventions only a limited number of times, while in some other countries there was more acceptance or repeated imposition of control measures during the COVID-19 pandemic. I certainly don't think it is incumbent on the authors to wade into all such thorny issues, but I think it would be worthwhile for them to acknowledge they exist and that their analysis represents only a first step in figuring out how to better optimize responses. [I now see that this was addressed a bit in the last paragraph, but still think it warrants a bit more discussion here].

Thanks, this is an excellent suggestion. We have now added more nuance in that paragraph of the Discussion to show awareness of the complex variables that must be weighed in making policy decisions and to indicate that our work is only a first step in better understanding and analysing the potential trade-offs among early responses and false alarms. We have also included more uncertainty in our language whenever proposing conservative approaches (e.g., see new Author Summary).

6 Discussion, "…enhancing syndromic surveillance…."
See above comments about limiting to syndromic.

We have edited this in accordance with our response to 4 above.

**Reviewer #2:**

In "Fundamental limits on inferring epidemic resurgence in real time using effective reproduction numbers" by Parag and Donnelly, the authors adopt a renewal model for epidemic progression and consider inference on the time-varying reproduction number $R_{s}$ in a growing/resurging or declining/controlled epidemic. The authors argue that there is an inherent asymmetry in the ability to detect a growing epidemic in real-time that arises due to the limited information that can be extracted from low case numbers. Parag & Donnelly argue that even with state-of-the-art Bayesian optimisation in practice this means that there is a lag of around a generation time before a shift to $R_{s}$ exceeding its threshold value can be detected, even with perfect case reporting, for a selection of 5 pathogens with different generation times. The authors further demonstrate that this delay in detecting resurgence is exacerbated by aggregating data across any heterogeneity.

This manuscript makes some interesting points that are important considerations for developing surveillance systems that aim to detect early outbreaks. The discussion of alternative and composite surveillance techniques is also valuable.

Many thanks for the positive appraisal.

Some minor points below:

As noted, the asymmetry in detecting resurgence is due to the assumption that if $R_{s}$ rises above 1 this is likely at a time when the epidemic is small (with small $\Lambda_{\tau(s)}$). During the SARS-CoV-2 pandemic we have sometimes seen $R_{s}$ increase above at a time when the epidemic is large, so it is perhaps worth noting that this less likely combination of $\lambda_{\tau(s)}$ and ${\Delta\lambda}_{\tau(s)}$ may still occur, and discuss whether case detection could still provide a relatively efficient and valuable route to inference of $R_{s}$ in these situations?

This is an excellent point. If a resurgent change occurs when case numbers are large then we would be able to detect this much more swiftly than an equivalent change at low case incidence. We have included a new paragraph in the Discussion explaining this point. We have also added the nuance that if incidence is large but the rise in *R* is localised to some group (e.g., a variant of concern that emerged) then the fundamental delays we uncovered still hold as the cases of that group will, at least initially, be small and the incidence contributed from other groups will act as background noise (Fig 4 essentially describes these types of dynamics) .

The decision to explore inference of $R_{s}$ in the case of perfect case detection is well motivated. As I understand it this analysis also assumes perfect knowledge of the generation interval distribution $w_{u}$, which may change over time for many reasons. Changes in $R_{s}$ due to shifts in behaviour (Ali et al., 2020) or due to emergence of new variants (Hart et al., 2021) may in particular be accompanied by changes in $w_{u}$. It could be interesting to discuss whether the asymmetry in sensitivity between detection of control and resurgence would still be significant when considering other such sources of uncertainty.

Ali et al., 2020, <https://pubmed.ncbi.nlm.nih.gov/32694200/>

Hart et al., 2021, <https://www.medrxiv.org/content/10.1101/2021.10.21.21265216v1>

We chose not to explore variations in the generation time distribution as those are generally not known in real time and often require non-routine surveillance data (e.g., studies exposing these changes tend to apply retrospective analysis on paired contact tracing data). However, the point is an interesting one and we have added text in the Methods on this assumption and to indicate how such a change would be accommodated. Additionally, we have commented on the likely impact of such changes on our results in the Discussion. We expect the asymmetry we found to be relatively robust though its magnitude might depend on when (relative to the *R* change-point) the change in generation time occurs. This is because we consistently found the detection lag to be 5-10 times longer for resurgence than control across pathogens with diverse generation time distributions. This is larger than the size of the relative change in generation times that is often reported in studies characterising varying generation time distributions.
